# A Simple and Low-Cost CRISPR/Cas9 Knockout System Widely Applicable to Insects

**DOI:** 10.3390/insects15050339

**Published:** 2024-05-08

**Authors:** Jun Cao, Keli Wu, Xin Wei, Jiaojiao Li, Chun Liu, Tingcai Cheng

**Affiliations:** State Key Laboratory of Resource Insects, Southwest University, Chongqing 400716, China; junc96@163.com (J.C.); wkl26622662@163.com (K.W.); weixwwx@163.com (X.W.); lijiaojiao942333@163.com (J.L.); mlliuchun@163.com (C.L.)

**Keywords:** CRISPR/Cas9, knockout, sgRNA, detailed method, insects, silkworm

## Abstract

**Simple Summary:**

Editing (deletion, insertion, and substitution) of target genes is a commonly used method for studying gene function, which has significant applications in disease treatment, agricultural improvement, and environmental protection. The CRISPR/Cas9 system is currently the most widely used gene-editing technology due to its low cost and high efficiency. Continuous improvements in this system by researchers have resulted in a wide range of applications and greatly advanced gene function research. However, many CRISPR/Cas9 systems are complex and difficult to share, limiting their use to a few laboratories. In this study, we present a simple, low-cost, and universal CRISPR/Cas9 knockout system. We provide detailed information on the plasmid sequences, reagent codes, and methods. This study can help researchers establish gene knockout systems and facilitate better gene function research.

**Abstract:**

The CRISPR/Cas9 gene-editing system is a standard technique in functional genomics, with widespread applications. However, the establishment of a CRISPR/Cas9 system is challenging. Previous studies have presented numerous methodologies for establishing a CRISPR/Cas9 system, yet detailed descriptions are limited. Additionally, the difficulties in obtaining the necessary plasmids have hindered the replication of CRISPR/Cas9 techniques in other laboratories. In this study, we share a detailed and simple CRISPR/Cas9 knockout system with optimized steps. The results of gene knockout experiments in vitro and in vivo show that this system successfully knocked out the target gene. By sharing detailed information on plasmid sequences, reagent codes, and methods, this study can assist researchers in establishing gene knockout systems.

## 1. Introduction

The Clustered Regularly Interspaced Short Palindromic Repeats/CRISPR-associated system (CRISPR/Cas) is a widely applicable gene-editing tool extensively employed in the study of gene function across various species [1,2,3,4,5,6]. The CRISPR/Cas system consists of two main components: single-guide RNA (sgRNA) and Cas protein. SgRNA is composed of a targeted sequence of 20 nt and approximately 80 nt of CRISPR RNA-Trans-activating CRISPR RNA (crRNA-tracrRNA) sequences, which is mainly used to guide the binding of Cas protein to the target region. According to the sequences that can be specifically recognized, Cas proteins are classified into Cas9, Cas12, Cas13, and Cas14, with Cas9 being the most widely used Cas protein [7]. After binding with crRNA-tracrRNA, the Cas9 protein targets the PAM sequence (NGG) of the gene and unwinds the DNA duplex. Then, the targeted sequence of sgRNA binds to the target site based on the principle of complementary base pairing, guiding the Cas9 protein to cleave double-stranded DNA [4,8]. Subsequently, the cells initiate non-homologous end joining (NHEJ) or homology-directed repair (HDR) to repair the broken DNA. This repair effect may lead to the loss, insertion, or alteration of bases, thereby causing genetic changes [9].

Nowadays, the CRISPR/Cas9 system has diversified applications. By integrating the region-specific promoter and piggyBac transposable system, specific gene knockout of different organ regions is achieved [10,11]. The Receptor-Mediated Ovary Transduction of Cargo (ReMOT) method has been employed for germline-level gene editing, which is an effective method to avoid embryo injection [12,13,14]. In addition, sgRNA can guide dCas9 to fuse with gene transcription activation domains, thereby promoting gene transcription [15]. Most CRISPR/Cas9 systems are complex, and plasmid sequences and implementation details cannot be shared, limiting their use to a small number of laboratories. For laboratories that have not established a knockout system, there is an urgent need for a simple and effective gene knockout system to provide a reference.

Here, we share a simple, low-cost, and universal CRISPR/Cas9 knockout system. The steps of this knockout system were optimized to reduce costs and improve efficiency. Further, we provide detailed plasmid sequences, reagent codes, and methods to assist researchers in establishing gene knockout systems.

## 2. Materials and Methods

### 2.1. Select Target Sequence of Gene and Design Primers

The target sequence is selected online at https://cctop.cos.uni-heidelberg.de/ (accessed on 8 October 2019), and the selection principle refers to previous reports [16]. After that, primers are designed for polymerase chain reaction (PCR). The composition of the forward primer is T7 promoter + GG + target sequence + the first 20 bases of the crRNA-tracrRNA—for example, TAATACGACTCACTATA + GG + TTGTGCAAATAGCATTCGTG + GTTTTAGAGCTAGAAATAGC. The sequence of reverse primer is constant, which is AAAAAAAGCACCGACTCGGTGCCAC. Primer sequences were listed in Table S1.

Notes: 1. High-specificity target sequences should be selected to prevent off-targeting. 2. For general gene knockout studies, we suggest selecting target sequences on the first exon, which can silence the gene by generating frameshift mutations. 3. If the first base of target sequence is guanine (G), there is no need to add GG after the T7 promoter. 4. The length of the target sequence is 20 bases. Do not add the PAM sequence (NGG) to the primer sequence. 5. We suggest selecting 2–3 target sequences per gene to increase the probability of splicing genes.

### 2.2. PCR Amplification of Transcriptional Templates

The reaction components of PCR are shown in Table 1. PCR program: 95 °C 5 min → 95 °C 10 s → 55 °C 15 s → 72 °C 15 s → 72 °C 7 min → 16 °C ∞. Steps 2–4 perform 38 cycles. Take 3 µL PCR product for gel electrophoresis detection. If the bands are clear and single, the recovery of nucleic acid fragments can be carried out.

Notes: 1. The above PCR system is for reference only. A general PCR system can be used to amplify 120 bp fragments, then perform in vitro transcription.

### 2.3. Recovery of Nucleic Acid Fragments (Ethanol Precipitation Method)

Use nuclease-free water to supplement the sample to 100 µL. Add 250 µL anhydrous ethanol, 10 µL 3 M sodium acetate, and 1 µL of acryl carrier. Let stand on ice for 1–2 h.

Centrifuge at 12,000× *g* for 5 min at 4 °C.

Discard the supernatant. Add 1 mL 75% ethanol and invert the tube 6–8 times.

Centrifuge at 12,000× *g* for 5 min at 4 °C.

Discard all the supernatant, open the tube cover, and let stand for 3 min.

Add 15 µL nuclease-free water to dissolve the precipitation. Measure the concentration.

Notes: 1. When using the ethanol precipitation method to recover PCR products, the acryl carrier must be added to promote nucleic acid precipitation. Be careful and do not pour out the precipitation. 2. To save costs, we provide the ethanol precipitation method for purifying PCR products. The gel recovery method can also be used, and we recommend the E.Z.N.A^®^ Gel Extraction Kit (D2500-03, OMEGA, Norcross, GA, USA). For detailed information, please refer to the manual. 3. The ethanol precipitation method cannot separate nucleic acid fragments of different sizes. If the PCR product is not a single band, please use the gel recovery method.

### 2.4. In Vitro Transcription

The purified PCR product is used as a template for in vitro transcription. The T7 RiboMAX™ Express Large Scale RNA Production System (P1320, Promega, Madison, WI, USA) is used to synthesize the sgRNA.

The reaction components of in vitro transcription are shown in Table 2. Incubate at 37 °C for 2 h.

Add 1 µL RQ1 RNase-Free DNase (Promega), and incubate at 37 °C for 15 min.

Incubate at 75 °C for 10 min, then immediately place it on ice for ≥5 min.

For detailed information on the purification of sgRNA using the ethanol precipitation method, please refer to step 2.3.

Measure the concentration of sgRNA and perform gel electrophoresis detection.

Notes: 1. After in vitro transcription, a large amount of RNA can be obtained, so the acryl carrier can be saved when using the ethanol precipitation method to recover RNA. 4. The complete sgRNA obtained through the above steps may have two bands, as the entangled RNA has not been fully unraveled. Just perform the annealing process again (incubate at 75 °C for 10 min, then immediately place it on ice for ≥5 min). 5. Qualified sgRNA bands must be clear, bright, and nondegradable.

### 2.5. In Vitro Knockout Detection

Design primers and perform PCR to amplify DNA fragments containing the target sequence. The appropriate fragment size is 500–800 bp (PCR system and program reference step 2.1, appropriate extension time needs to be changed). Use the ethanol precipitation method for DNA fragment recovery (step 2.3) and use it as target DNA.

The reaction components are shown in Table 3. Incubate at 37 °C for 2 h. After that, incubate at 65 °C for 10 min and perform gel electrophoresis detection.

Notes: 1. For research groups that are establishing a knockout system for the first time based on this article, we strongly recommend conducting in vitro knockout detection to verify the correctness of this system. 2. For research groups that have already matured the use of this system, this step can be omitted. In our experience, all sgRNAs can successfully cleave target DNA in vitro.

### 2.6. Establishment of Knockout Line

Mix SgRNAs and Cas9 protein at a molar ratio of approximately 2:1 for embryo injection. For the silkworm, inject a mixture of 3–5 nL into embryos within 2 h of eggs being laid. Then, seal the wound with quick-drying glue and incubate the embryo in an appropriate environment.

The hatched larvae are reared into adults and named Generation 0 (G0); Generation1 (G1) eggs are obtained via random intercross breeding of G0 adults. Randomly select 20 G1 eggs from each parent and extract the genome using DNAiso regent, as described in the manual. Design primers containing sgRNA and perform PCR (primers for amplifying target DNA in step 2.5 can be used). The PCR products are sequenced.

Analyze the sequencing results and select the eggs that produce overlapping peaks at the target site. Hatch the remaining eggs and rear them to larva. Randomly select a certain number of larvae, extract the genome from each larva, and perform PCR. Sequence the PCR products. After confirming the knockout form of G1 individuals, carry out a phenotype survey, molecular detection, and germplasm preservation.

Notes: 1. The screening of knockout lines shows little difference among different insects. We provide a strategy for screening knockout lines in silkworm, which scholars can refer to. 2. For the ratio of sgRNA and Cas9 protein mixture, we recommend mixing 10 µL sgRNA and 1 µg Cas9 protein. For embryos of different sizes, it is recommended to change the injection volume. 3. We suggest injecting the sgRNA and Cas9 protein mixture as soon as possible after egg laying to improve knockout efficiency. 4. Given the goal of obtaining heritable mutant lines and improving efficiency, knockout detection of G0 is unnecessary. 5. Wild type is required as a control for all PCR and sequencing analyses.

## 3. Results

### 3.1. Optimization of sgRNA Synthesis Steps

**Figure 1:**
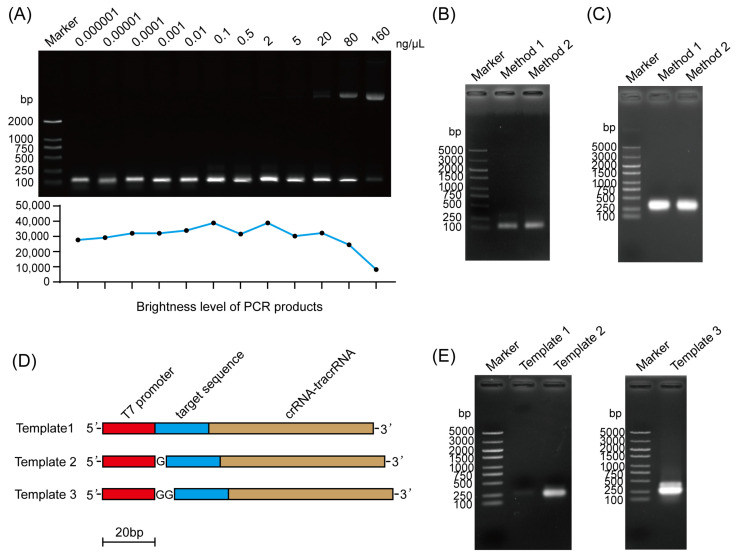
Optimization of sgRNA synthesis. (**A**) PCR analysis using different concentrations of T_PMD19_-sgRNA plasmids. The brightness level of PCR products was digitized. (**B**,**C**) Analysis of transcription template (**B**) and sgRNA (**C**) obtained by two methods. Method 1, gel recovery method; Method 2, ethanol precipitation method. (**D**) Schematic structure of template 1, template 2, and template 3 for in vitro transcription. G, guanine. Scale bar, 20 bp. (**E**) Analysis of sgRNAs synthesized with different templates.

To reduce costs and improve efficiency, we optimized the steps of sgRNA synthesis. Firstly, the optimal template concentration for PCR was explored in the range of 0.000001 ng/μL–160 ng/μL. The results show that as the T_PMD19_-sgRNA plasmid concentration increased, the amount of PCR products first increased and then decreased. At the plasmid concentration of 2 ng/µL, the amount of PCR products was the highest (Figure 1A). This indicated that the optimal concentration of T_PMD19_-sgRNA plasmid for PCR was 2 ng/µL. After that, the feasibility of using the ethanol precipitation method to recover PCR products was evaluated. The PCR products could be recovered by the ethanol precipitation method, and there was no difference compared with the gel recovery method (Figure 1B). Further in vitro transcription results showed no difference in sgRNA obtained by these two methods (Figure 1C). This indicates that the ethanol precipitation method could be used to replace the gel recovery method, thereby reducing costs. Several scholars have emphasized that the guanine at the 3′ end of the T7 promoter was very important for the transcription of T7 RNA polymerase [17,18]. To verify this conclusion, different primers were designed. The transcription template was amplified by PCR, and then the efficiency of in vitro transcription was detected (Figure 1D). The results show that compared with temple 1 and temple 2, temple 3 achieved the highest transcription efficiency (Figure 1E). The above results indicate that two guanines should be added at the 3′ end of the T7 promoter when designing primers to improve the transcription efficiency in vitro.

### 3.2. Feasibility Verification of the CRISPR/Cas9 System

**Figure 2:**
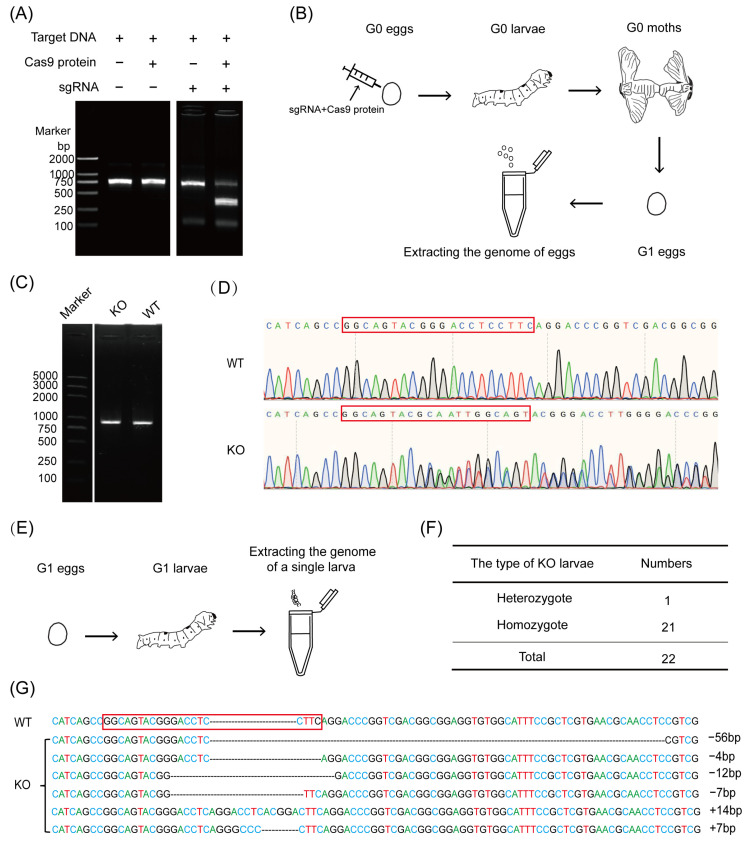
Feasibility analysis of the CRISPR/Cas9 knockout system. (**A**) In vitro feasibility analysis of the CRISPR/Cas9 system. The figure is spliced from different areas on the same gel. (**B**) Schematic diagram of knockout line screening. G0, Generation 0. G1, Generation 1. (**C**) Detection of PCR products from KO and WT lines. KO, knockout. WT, wild type. The figure is spliced from different areas on the same gel. (**D**) Sequencing of PCR products from KO and WT lines. The red box marks the target site. (**E**) Schematic diagram of G1 larval detection. (**F**) Number of homozygotes and heterozygotes in G1 larvae. (**G**) Genomic sequences around the sgRNA in KO and WT lines of G1. The red box marks the target site.

To verify the feasibility of this CRISPR/Cas9 system, in vitro knockout experiments were performed using DNA containing the target site (target DNA). The results show that after sgRNA and cas9 protein were combined, the target DNA was successfully sheared, resulting in smaller nucleic acid fragments (Figure 2A). This indicates that this CRISPR/Cas9 system can shear the target DNA in vitro. After that, the in vivo knockout experiment was carried out in the silkworm, *Bombyx mori*. SgRNA and cas9 protein were mixed and injected into silkworm eggs. The hatched larvae (Generation 0, G0) were reared to adults, and Generation 1 (G1) eggs were obtained by random intercross breeding. G1 eggs were randomly selected from each parent to extract the genome (Figure 2B). Using the genome as a template, PCR was used to amplify genomic fragments containing target sequences. The electrophoretic detection results showed no difference in PCR products between the knockout and wild-type lines (Figure 2C). Subsequently, the PCR products were sequenced, and it was found that the knockout line produced overlapping peaks at the target site, while the sequencing profile of the wild type was normal (Figure 2D). These results indicate that this CRISPR/Cas9 system successfully sheared the target gene in vivo and generated base changes at the target site. To further confirm the knockout type of G1, the remaining eggs were incubated, and the genomes of individual larvae were extracted (Figure 2E). The sequencing results of 22 samples showed that 1 sample was heterozygous for overlapping peaks at the target sequence and 21 samples were homozygous for normal peaks (Figure 2F). Further alignment of the sequences of homozygotes revealed that the knockout lines generated the insertion and deletion of 4–56 bp of bases at the target site (Figure 2G). The above results show that this CRISPR/Cas9 system could obtain effective knockout lines and that the majority of G1 larvae were homozygous.

## 4. Discussion

To make better use of this CRISPR/Cas9 system, the detailed method is presented in this article. The crRNA-tracrRNA sequence is displayed in the profile of the T-vector PMD19 plasmid (Figure S1), allowing researchers to construct the same plasmid base on this profile. Reagent codes are also listed in Table S2 for easy reference and procurement. Furthermore, a detailed experimental protocol including sgRNA synthesis, in vitro knockout, and establishment of the knockout line is provided, and notes for each step are highlighted.

This is a simple and detailed CRISPR/Cas9 knockout system, but there are still limitations. This system is only suitable for whole-body gene knockout and must be assisted by embryo injection equipment. In addition, for some special genes, such as lethal genes and reproductive genes, it was difficult to explore the gene function because of the inability to obtain heritable lines. We found that some genes produced knockout phenotypes in G0. Drawing on the fact that RNA interference (RNAi) is widely used in contemporary gene knockdown [19,20,21], we believe that this CRISPR/Cas9 system is also suitable for contemporary gene function research.

## Figures and Tables

**Table 1:** The reaction components of PCR.

Component	Quantities
10× PCR buffer/µL	5
dNTP/µL	4
TaKaRa Taq™/µL	0.5
Primer F (2 µM)/µL	5
Primer R (2 µM)/µL	5
T_PMD19_-sgRNA plasmid/ng	100
Nuclease-free water/µL	Up to 50

**Table 2:** The reaction components of in vitro transcription.

Component	Quantities
T7 transcription 5× buffer/µL	4
rNTP/µL	6
Template/µg	1
Enzyme mix/µL	2
Nuclease-free water/µL	Up to 20

**Table 3:** The reaction components of in vitro knockout detection.

Target DNA	sgRNA (1000 ng/µL)	BSA (0.5 mg/mL)	NE Buffer3	Cas9 Protein	Nuclease-Free Water
200 ng	0	2 µL	1 µL	0	Up to 10 µL
200 ng	0	2 µL	1 µL	0.5 µg	Up to 10 µL
200 ng	1 µL	2 µL	1 µL	0	Up to 10 µL
200 ng	1 µL	2 µL	1 µL	0.5 µg	Up to 10 µL

## Data Availability

The original contributions presented in the study are included in the article/Supplementary Materials, further inquiries can be directed to the corresponding author.

## Supplementary Materials

The following supporting information can be downloaded at: https://www.mdpi.com/article/10.3390/insects15050339/s1, Table S1: Primer sequences used in this study; Table S2: The reagents used in this study; Figure S1: The profile of the T_PMD19_-sgRNA plasmid.
