# Supplementary material for: A Simple and Low-Cost CRISPR/Cas9 Knockout System Widely Applicable to Insects
Source: Insects. 2024 May 8;15(5):339. doi: 10.3390/insects15050339 (PMC11122616; doi:10.3390/insects15050339)

Table S1 Primer sequences used in this study

Primer	Sequence
Template 1-F	TAATACGACTCACTATATTGTGCAAATAGCATTCGTGGTTTTAGAGCTAGAAATAGC
Template 2-F	TAATACGACTCACTATAGTTGTGCAAATAGCATTCGTGGTTTTAGAGCTAGAAATAGC
Template 3-F	TAATACGACTCACTATAGGTTGTGCAAATAGCATTCGTGGTTTTAGAGCTAGAAATAGC
sgRNA-R	AAAAAAAGCACCGACTCGGTGCCAC
Target DNA-F	ATGCCACACTGGGTAACTGCTG
Target DNA-R	GAAGAAACCTTGCGTCCGTGT
KO-Test-F	ACCTCACCGAAGTTAGTTACG
KO-Test-R	TTTGGACAGGTGTCAAATGGG

Table S2 The reagents used in this study

Reagent	Company	Code
10×PCR Buffer (Mg^{2+} free)	TAKARA	9151AM
TAKARA Taq™	TAKARA	R001B
dNTP	TAKARA	4030Q
3M Sodium acetate	Promega	P135A
Acryl Carrier	Solarbio	SA1020
Guide-it™ Recombinant Cas9 (10 μg/μL)	TAKARA	632679
E.Z.N.A® Gel Extraction Kit	Omega	D2500-03
T7 RiboMAX™ Express Large Scale RNA Production System	Promega	P1320
DNAiso Regent	TAKARA	9770A
NE Buffer3	New England BioLabs	B7003S
T-Vector PMD™19 (Simple)	TAKARA	3271

Figure S1 The profile of the T$_{PMD19}$-sgRNA plasmid.

Lac Z ⟶ M13 F

CGTATGTTGTGTGGAATTGTGAGCGGATAACAATTTCACACAGGAAACAGCTATGACCATGATTAC
GCATACAACACACCTTAACACTCGCCTATTGTTAAAGTGTGTCCTTTGTCGATACTGGTACTAATG

GCCAAGTTTGCACGCCTGCCGTTCGACGATTGTTTTAGAGCTAGAAATAGCAAGTTAAAATAAGGC
CGGTTCAAACGTGCGGACGGCAAGCTGCTAACAAAATCTCGATCTTTATCGTTCAATTTTATTCCG

crRNA-tracrRNA (83bp)

TAGTCCGTTATCAACTTGAAAAAGTGGCACCGAGTCGGTGCTTTTTTTAATCTCTGGAAGATCCGC
ATCAGGCAATAGTTGAACTTTTTCACCGTGGCTCAGCCACGAAAAAAATTAGAGACCTTCTAGGCG

GCGTACCGAGTTCTAATTCACTGGCCGTCGTTTTACAACGTCGTGACTGGGAAAACCCTGGCGTTA
CGCATGGCTCAAGATTAAGTGACCGGCAGCAAAATGTTGCAGCACTGACCCTTTTGGGACCGCAAT

M13 R

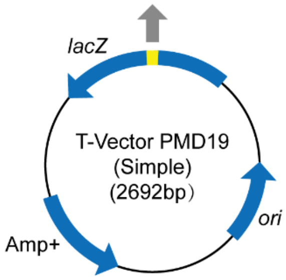

Supplement: Supplementary file 1 [file insects-15-00339-s001.zip › insects-2978222-supplementary.pdf]
